# Phaseless Terahertz Coded-Aperture Imaging Based on Incoherent Detection

**DOI:** 10.3390/s19020226

**Published:** 2019-01-09

**Authors:** Long Peng, Chenggao Luo, Bin Deng, Hongqiang Wang, Yuliang Qin, Shuo Chen

**Affiliations:** School of Electronic Science, National University of Defense Technology, Changsha 410073, China; penglong_nudt@163.com (L.P.); dengbin_nudt@163.com (B.D.); oliverwhq@tom.com (H.W.); youngqin@126.com (Y.Q.); chenshuo_nudt@163.com (S.C.)

**Keywords:** terahertz, coded-aperture imaging, phaseless imaging, incoherent detection, phase retrieval

## Abstract

In this paper, we propose a phaseless terahertz coded-aperture imaging (PTCAI) method by using a single incoherent detector or an incoherent detection array. We at first analyze and model the system architecture, derive the matrix imaging equation, and then study the phase retrieval techniques to reconstruct the original target with high resolution. Numerical experiments are performed and the results show that the proposed method can significantly reduce the system complexity in the receiving process while maintaining high resolution imaging capability. Furthermore, the approach of using incoherent detection array instead of single detector is capable of decreasing the encoding and sampling times, and therefore helps to improve the imaging frame rate. In our future research, the method proposed in this paper will be experimentally tested and validated, and high-speed PTCAI at nearly real-time frame rates will be the main work.

## 1. Introduction

Recently, terahertz imaging technologies have attracted increasing attention in areas such as security checks, terminal guidance, and battlefield reconnaissance [1]. Terahertz waves have higher frequencies and shorter wavelengths than microwaves and millimeter waves, thus THz imaging systems have larger bandwidths and higher resolution in both azimuth and range directions. Moreover, terahertz waves have stronger penetration than light waves in imaging. Based on the basic principles of both optical coded-aperture imaging [2,3] and radar correlation imaging [4,5], a terahertz coded-aperture imaging (TCAI) [6,7,8] system can modulate the incident THz wave and generate a spatiotemporal uncorrelated wave distribution with an electronic coded aperture. By modeling the imaging system and imaging process, a multi-parameter imaging equation can be obtained and, therefore, the target scattering coefficients can be reconstructed according to the computational imaging method. By contrast with traditional electromechanical scanning radar [9,10] and synthetic aperture radar [11,12,13], TCAI can achieve high resolution, a high frame rate and forward looking without relying on any relative motion between the radar and the target.

Nowadays, accurate measurements of the phase still face great challenges in terahertz band [14,15]. For a traditional coherent detection TCAI system, high frequency and large bandwidth are beneficial to high-resolution imaging in azimuth and range dimensions. However, the coherent detection of high-frequency and large-bandwidth THz signals requires complex physical receiving chains for down-conversion or bandwidth reducing, which will necessarily add cost in terms of size, power, and hardware. Especially for the TCAI system working with frequency-hopping signals, it would be quite intractable since the dechirping method [16] cannot be adopted to process the echo signal.

Accordingly, we propose a phaseless terahertz coded-aperture imaging (PTCAI) method based on incoherent detection techniques. The proposed method utilizes a single incoherent detector [17] or an incoherent detection array [18] to record directly the intensity responses of the echo signal, and it does not require any down-conversion or bandwidth reducing components. Here, the incoherent detectors should be properly designed to work in the THz band and cover the operating frequencies of the PTCAI system. The sampled intensity data can be used to build the matrix imaging equation and the target can be reconstructed by using the phase retrieval techniques [19,20,21,22] according to computational imaging theory. Therefore, the incoherent detection techniques have helped to make the PTCAI system more compact and cost-effective than a traditional TCAI system.

Additionally, the TCAI method with a single detector requires large amount of sampling and encoding times to obtain enough information for reconstructing the target. The approach of using a detector array instead of a single detector will significantly reduce the measuring times [23]. Here, measuring refers to the encoding of the echo signal and sampling of the modulated signal. Thus, it is possible to realize a novel TCAI system working with fewer frequency points and higher imaging speed, and it is more suitable for recording and imaging moving targets.

This paper is organized as follows. In Section 2, the PTCAI system configuration is first introduced while the matrix imaging equations are created in detail. Section 3 analyzes the target reconstruction principle for PTCAI and introduces a phase retrieval algorithm for computational imaging. In Section 4, numerical experiments are implemented and results demonstrate the feasibility of the PTCAI system based on incoherent detection. Finally, the main work and contributions of this paper are summarized in Section 5.

## 2. System Configuration and Matrix Imaging Equation

### 2.1. Phaseless Terahertz Coded-Aperture Imaging (PTCAI) System with Single Incoherent Detector

**Figure 1:**
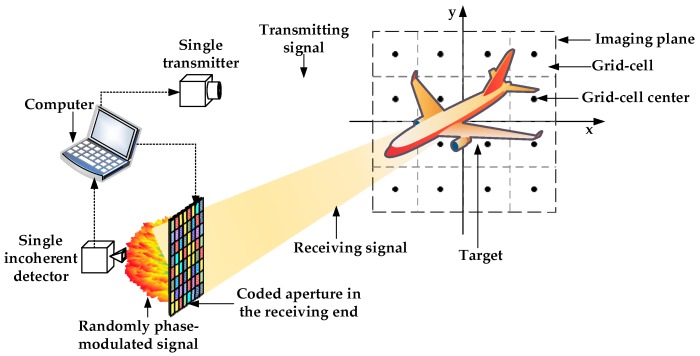
The architecture of phaseless terahertz coded-aperture imaging (PTCAI) system with single incoherent detector.

Receivers used in conventional imaging systems can receive echo signals carrying both phase and amplitude information. This contributes to the signal processing and target reconstruction at the expense of complex receiving chains and expensive components. In our design, a more compact and cost-effective approach has been proposed in which a single incoherent detector instead of a conventional coherent receiver is used to receive the intensity information of the echo signal. As shown in Figure 1, the proposed PTCAI system mainly contains a single transmitter, a single incoherent detector, and a coded aperture in the receiving end. The THz wave is transmitted by the transmitter and propagated to the imaging plane. The imaging plane is divided into *W* grid-cells where the center position vector of each grid cell is $r_{w}$, and the strong scatterers of target are assumed to be located at the center of each grid-cell exactly. The amplitude or phase of the echo signal can be modulated randomly or pseudo-randomly by a computer-controlled electronic coded aperture [24]. The spatiotemporal uncorrelated THz field is then detected by the single incoherent detector and only the intensity information is recorded and transmitted to the computer for further processing and imaging.

For simplicity, in describing the imaging process, we establish the mathematical signal model of the PTCAI system with single incoherent detector. Suppose a single transmitter emits a terahertz linear frequency modulation (LFM) signal, which can be written as,

(1) $$S_{t}(t)=A\exp\left[j2\pi \left(f_{c}t+\frac{K}{2}t^{2}\right)\right],$$

where $S_{t}(t)$ is the transmitting signal at time t, $f_{c}$ is the carrier frequency, *A* is the amplitude of the signal, and *K* is the chirp rate. Then, the reference signal $S(t_{n},r_{w})$ of the *w*-th grid cell at time $t_{n}$ can be expressed as

(2) $$S(t_{n},r_{w})=\sum_{q=1}^{Q}A\exp\left(j2\pi \left[f_{c}\left(t_{n}-\frac{\tau }{c}\right)+\frac{K}{2}{\left(t_{n}-\frac{\tau }{c}\right)}^{2}\right]+\varphi_{q}\left(t_{n}\right)\right).$$

The propagation distance of the echo signal is $\tau =\left|r_{w}-r_{tra}\right|+\left|r_{rec}-r_{q}\right|+\left|r_{q}-r_{w}\right|$, where $r_{w}$, $r_{tra}$, $r_{rec}$ and $r_{q}$ are position vectors of the *w*-th grid cell, the transmitter, the receiver and the *q*-th coded aperture element, respectively. And $\varphi_{q}(t_{n})$ is the random phase modulation factor of the *q*-th coding aperture element at time $t_{n}$. Hence, the received signal at time $t_{n}$ is expressed as

(3) $$Sr(t_{n})={\left|\sum_{w=1}^{W}\beta_{w}S\left(t_{n},r_{w}\right)\right|}^{2},$$

where $\beta_{w}$ is the scattering coefficient corresponding to the *w*-th grid cell, and |·| denotes the absolute value. Thus, the matrix imaging equation is written as,

(4) $$\left[\begin{array}{c} Sr(t_{1})\\ Sr(t_{2})\\ \vdots \\ Sr(t_{N}) \end{array}\right]={\left|\left[\begin{array}{cccc} S(t_{1},r_{1}) & S(t_{1},r_{2}) & \cdots & S(t_{1},r_{W})\\ S(t_{2},r_{1}) & S(t_{2},r_{2}) & \cdots & S(t_{2},r_{W})\\ \vdots & \vdots & \cdots & \vdots \\ S(t_{N},r_{1}) & S(t_{N},r_{2}) & \cdots & S(t_{N},r_{W}) \end{array}\right]\cdot \left[\begin{array}{c} \beta_{1}\\ \beta_{2}\\ \vdots \\ \beta_{W} \end{array}\right]+\left[\begin{array}{c} \omega_{1}\\ \omega_{2}\\ \vdots \\ \omega_{N} \end{array}\right]\right|}^{2},$$

where ${\left\{\omega_{n}\right\}}_{n=1}^{n=N}$ is the additive measurement noise. The above formula can also be written concisely as,

(5) $$Sr={\left|S\beta +\omega \right|}^{2},$$

where $Sr={\left[Sr(t_{1}),Sr(t_{2}),\ldots ,Sr(t_{N})\right]}^{T}$ and $\beta ={\left[\beta_{1},\beta_{2},\ldots ,\beta_{W}\right]}^{T}$ are echo signal vector and scattering coefficient vector, respectively. $\omega ={\left[\omega_{1},\omega_{2},\ldots ,\omega_{N}\right]}^{T}$ is the measurement noise vector, and ${\left[\cdot \right]}^{T}$ denotes matrix transposition. S is the reference signal matrix, and can be expressed as

(6) $$S=\left[\begin{array}{cccc} S(t_{1},r_{1}) & S(t_{1},r_{2}) & \cdots & S(t_{1},r_{W})\\ S(t_{2},r_{1}) & S(t_{2},r_{2}) & \cdots & S(t_{2},r_{W})\\ \vdots & \vdots & \cdots & \vdots \\ S(t_{N},r_{1}) & S(t_{N},r_{2}) & \cdots & S(t_{N},r_{W}) \end{array}\right].$$

Obviously, the time-domain samples at ${\left\{t_{n}\right\}}_{n=1}^{n=N}$ corresponding to each grid cell and the spatial-domain samples on ${\left\{r_{w}\right\}}_{w=1}^{w=W}$ corresponding to each sampling time can be represented by the column vectors and row vectors of S, respectively, where the correlation between different columns and rows is randomly modulated by the coded aperture.

### 2.2. PTCAI System with Incoherent Detector Array

**Figure 2:**
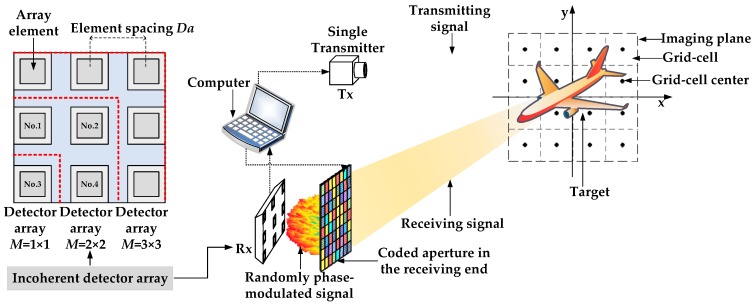
The architecture of the PTCAI system with incoherent detector array.

Since the PTCAI system with single detector requires a large amount of sampling and number of encoding times to obtain enough information for reconstructing the target, we use an incoherent detector array to replace the single detector mentioned in Figure 1 above. The arrangement of array elements and the system configuration are shown in Figure 2. Each array element in the detector array can detect and transmit the intensity information of the echo signal to the computer independently. The correlations between signals detected by different array elements are affected by the element spacing *Da* which refers to the distance between two adjacent elements. In order to describe it correctly, we use M=e×e, e=1,2,3,… to represent an incoherent detector array containing e×e array elements, as shown in Figure 2.

The LFM signal is still used as the transmit signal. The center position vector of the *m*-th array element is $r_{m}$, and when the signal is received by the *m*-th array element at time $t_{n}$, the reference signal $S_{m}(t_{n},r_{w})$ of the *w*-th grid cell of the imaging plane can be expressed as,

(7) $$S_{m}(t_{n},r_{w})=\sum_{q=1}^{Q}A\exp\left(j2\pi \left[f_{c}\left(t_{n}-\frac{\tau_{m}}{c}\right)+\frac{K}{2}{\left(t_{n}-\frac{\tau_{m}}{c}\right)}^{2}\right]+\varphi_{q}(t_{n})\right),$$

where $\tau_{m}=\left|r_{w}-r_{tra}\right|+\left|r_{rec\left(m\right)}-r_{q}\right|+\left|r_{q}-r_{w}\right|$, as mentioned above, is the superimposed distance of the signal received by the *m*-th receiving element. The echo signal $Sr_{m}(t_{n})$ received by the *m*-th array element at time $t_{n}$ can be written as,

(8) $$Sr_{m}(t_{n})={\left|\sum_{w=1}^{W}\beta_{w}S_{m}(t_{n},r_{w})\right|}^{2}.$$

Hence, the signal detected by the entire array at time $t_{n}$ is represented as,

(9) $$Sr_{array}(t_{n})={\left[Sr_{1}(t_{n}),Sr_{2}(t_{n}),\ldots ,Sr_{m}(t_{n})\right]}^{T}.$$

Therefore, the entire matrix imaging equation can be expressed as,

(10) $$\left[\begin{array}{c} Sr_{array}(t_{1})\\ Sr_{array}(t_{2})\\ \vdots \\ Sr_{array}(t_{N}) \end{array}\right]={\left|\left[\begin{array}{cccc} S_{array}(t_{1},r_{1}) & S_{array}(t_{1},r_{2}) & \cdots & S_{array}(t_{1},r_{W})\\ S_{array}(t_{2},r_{1}) & S_{array}(t_{2},r_{2}) & \cdots & S_{array}(t_{2},r_{W})\\ \vdots & \vdots & \cdots & \vdots \\ S_{array}(t_{N},r_{1}) & S_{array}(t_{N},r_{2}) & \cdots & S_{array}(t_{N},r_{W}) \end{array}\right]\cdot \left[\begin{array}{c} \beta_{1}\\ \beta_{2}\\ \vdots \\ \beta_{W} \end{array}\right]+\left[\begin{array}{c} \omega_{1}\\ \omega_{2}\\ \vdots \\ \omega_{N} \end{array}\right]\right|}^{2},$$

where $S_{array}(t_{n},r_{w})$ is the entire reference signal of the all array elements related to the *w*-th grid cell at time $t_{n}$, and is written as,

(11) $$S_{array}(t_{n},r_{w})={\left[S_{1}(t_{n},r_{w}),S_{2}(t_{n},r_{w}),\ldots ,S_{M}(t_{n},r_{w})\right]}^{T}.$$

Equation (10) can be abbreviated as,

(12) $$Sr_{systerm}={\left|S_{systerm}\beta +\omega \right|}^{2},$$

where $Sr_{systerm}={\left[Sr_{array}(t_{1}),Sr_{array}(t_{2}),\ldots ,Sr_{array}(t_{N})\right]}^{T}$ is the signal detected by the entire array. $\beta ={\left[\beta_{1},\beta_{2},\ldots ,\beta_{W}\right]}^{T}$ and $\omega ={\left[\omega_{1},\omega_{2},\ldots ,\omega_{N}\right]}^{T}$ are the target scattering coefficient vector and the measurement noise vector, respectively. Therefore, the reference signal matrix of the PTCAI system with incoherent detector array is written as,

(13) $$S_{systerm}=\left[\begin{array}{cccc} S_{array}(t_{1},r_{1}) & S_{array}(t_{1},r_{2}) & \cdots & S_{array}(t_{1},r_{W})\\ S_{array}(t_{2},r_{1}) & S_{array}(t_{2},r_{2}) & \cdots & S_{array}(t_{2},r_{W})\\ \vdots & \vdots & \cdots & \vdots \\ S_{array}(t_{N},r_{1}) & S_{array}(t_{N},r_{2}) & \cdots & S_{array}(t_{N},r_{W}) \end{array}\right].$$

When the amount of information obtained is *L*, the encoding and sampling times are *N* = *L*/*M* by using an incoherent detector array while *N* = *L* by using a single incoherent detector. Thus, reducing the sampling and encoding times by increasing the number of array elements *M* is a viable option when obtaining the same amount of information.

## 3. Target Reconstruction Principle and Algorithms

The imaging equation of a conventional TCAI system with coherent detectors is generally written as $Sr_{TCAI}=S\beta +\omega$, and solving this linear equation is a convex problem for many reconstruction algorithms. For instance, the compressed sensing (CS) [25] theory can be adopted to achieve fast and high-resolution imaging in the conventional TCAI system with the advantage of using a small amount of detection data. The basis pursuit (BP) algorithm [26] and the orthogonal matching pursuit (OMP) algorithm [27] are more efficient when the target is sparse, while the total variation (TV) regularization, such as the TVAL3 [28] algorithm, is worth considering when the target is non-sparse. Nevertheless, the TVAL3 algorithm is also suitable for reconstructing sparse targets. However, the method of solving the imaging equation $Sr={\left|S\beta +\omega \right|}^{2}$ that we propose in the PTCAI system is actually a non-convex quadratic program, thus the reconstruction algorithms of the conventional TCAI system is no longer suitable.

To briefly describe this problem, it is assumed that the number of detection data obtained by the PTCAI system is L. Measurements and the *i*-th error term are $y_{i}=Sr_{i}={\left|S_{i}\beta +\omega_{i}\right|}^{2},i=1,2,\ldots ,L$ and $r_{i}≜y_{i}-{\left|S_{i}\beta \right|}^{2}$, respectively, where $S_{i}$ represents the *i*-th row vector of the reference signal matrix. Solving β from $Sr={\left|S\beta +\omega \right|}^{2}$ to reconstruct the target is our ultimate goal for the PTCAI approach. By defining 𝓁(x,y) as a squared loss function which can be written as $𝓁\left(x,y\right)={\left(x-y\right)}^{2}$, the problem of solving $Sr={\left|S\beta +\omega \right|}^{2}$ is transformed into,

(14) $$\begin{array}{cc} \mathrm{minimize}\;f(\beta ) & ≜\frac{1}{2L}\sum_{i=1}^{L}𝓁\left(y_{i},{\left|S_{i}\beta \right|}^{2}\right)=\frac{1}{2L}\sum_{i=1}^{L}r_{i}{}^{2}\\ & =\frac{1}{2L}{\sum_{i=1}^{L}\left({\left|S_{i}\beta \right|}^{2}-y_{i}\right)}^{2} \end{array}$$

In fact, the function f(β) is non-convex so that minimizing the f(β) is generally non-deterministic polynomial hard (NP-hard). But fortunately, the phase retrieval techniques are able to provide many novel and effective methods for solving the imaging equation $Sr={\left|S\beta +\omega \right|}^{2}$, and have attracted many researchers’ attention in recent years. Currently, the Wirtinger flow (WF) algorithm [29] and its improved algorithms, such as truncated Wirtinger flow (TWF) algorithm [30] and Wirtinger flow with optimal stepsize (WFOS) algorithm [31], can be applied to solve phase retrieval problems. The main step of the WF algorithm is to initialize the non-convex target by the spectral method, and then iteratively solve it by using the Winger gradient descent method. The TWF algorithm performs truncation initialization on the target and then uses the truncation gradient in iterative solutions. Although the TWF algorithm loses a certain precision, it has a faster convergence speed, fewer iterations, and shorter imaging time than the WF algorithm. The two algorithms mentioned above can effectively solve the imaging equation $Sr={\left|S\beta +\omega \right|}^{2}$ with empirical values of constant parameters. However, these heuristic rules are not the best settings, because too large a step leads to divergence in the iterative process while too small a step leads to slow convergence. In response to this problem, Jiang, X. et al. proposed the WFOS algorithm with the optimal step size $\alpha^{k}$ in [31]. Compared with the WF algorithm, the WFOS algorithm can obtain the same precision with fewer iterations, without relying on the empirical selection of constant parameters, and also can significantly shorten the imaging time.

## 4. Numerical Experiments and Discussion

In this section, the technical feasibility of PTCAI is demonstrated by numerical experiments. The experiment is performed on a computer with Intel Xeon CPU Gold 5188 (Intel Corporation, Santa Clara, CA, USA) at 2.3 GHz and 128 GB of memory. The WFOS algorithm is adopted in the process of solving the imaging equation while the parallel operation is used in the program to calculate the reference signal matrix S quickly. Both the TCAI system and the proposed PTCAI system consist primarily of a single transmitter, a detector array containing M=e×e, e=1,2,3,… array elements, and a transmission-type coded aperture. The size of the coded aperture is 0.5 m×0.5 m, with 30×30 elements for the echo signal’s phase modulation. The imaging plane is evenly divided into W=30×30 grid cells, each of which is 0.02 m×0.02 m. The single transmitter emits a terahertz LFM signal, and the basic parameter settings used in the numerical experiments are shown in Table 1. Therefore, in the frequency range of 334 GHz to 346 GHz, incoherent detectors used in the PTCAI system should be sufficiently sensitive to successfully detect the intensity of the echo signal, and in [32], the terahertz detector can provide both high sensitivity and high speed at this frequency range. The ratio between the number of detection data for imaging (*L*) and the number of grid cells in the imaging plane (*W*) can be written as *L/W*. The number of non-zero scattering coefficients in the imaging plane is considered the size of the target (*T*), and the ratio between *T* and *W* can be written as *T/W*.

Imaging performance is measured by the probability of successful imaging (PSI) and the relative imaging error (RIE) in the numerical experiments. RIE is defined as $\mathrm{RIE}=20\log_{10}\left({\left\|\tilde{\beta }-\beta \right\|}_{2}/{\left\|\beta \right\|}_{2}\right)$, where β and $\tilde{\beta }$ are the true and estimated scattering coefficient vectors [33], respectively. Here, ${\left\|\cdot \right\|}_{2}$ denotes the Euclidean norm. PSI is defined as ${\mathrm{PSI}=20\log_{10}\min{\left(\tilde{\beta }\right)}_{\Lambda }/\max\left(\hat{\tilde{\beta }}\right)}_{\Lambda }$, where Λ represents the set comprising the target scatterers, and ${\left(\tilde{\beta }\right)}_{\Lambda }$ takes the same values as $\tilde{\beta }$ at set Λ while ${\left(\hat{\tilde{\beta }}\right)}_{\Lambda }$ takes 0 at Λ and takes the same values as $\tilde{\beta }$ at every other indices [33,34]. Therefore, the success rate of imaging is in direct proportion to PSI, and reconstructing the target can be considered successful when PSI ≥ 0. It is obvious that the higher the PSI, the lower the RIE, which means that the imaging performance is better.

### 4.1. Comparisons between Conventional TCAI and PTCAI with Single Detector

To demonstrate the imaging performance of the PTCAI system with only intensity information, the conventional TCAI and the proposed PTCAI with single detector are both simulated to reconstruct the sparse target and the extended target. The scattering coefficients of the target ranges from 0 to 1, which makes it closer to the real target. In the experiment, the white Gaussian noise is added to the echo signal in the TCAI and PTCAI system, and the simulation setting of the signal-to-noise ratio (SNR) is SNR = 20 dB. The OMP and TVAL3 algorithms are adopted in TCAI to solve the imaging equation while the TWF and WFOS algorithms are adopted in PTCAI, and all reconstruction results are compared in detail. After obtaining the echo signals of TCAI or PTCAI, we adjust the parameters of all algorithms to get the best results in order to compare them reasonably. According to default values in [30], $\alpha_{z}^{lb}=0.3$, $\alpha_{z}^{ub}=\alpha_{h}=5$, and $\alpha_{y}=3$ are selected as parameters in TWF, and the step size α=0.001 is finally adopted through trial and error. This setting is based on recommendations of the article [30] and results of our repeated experiments, so it may not be the optimal setting. Since a truncated initialization operation is performed in the WFOS algorithm, it also needs to select a truncation parameter $\alpha_{y}=3$. In order to obtain stable reconstruction results and allow them to be compared within an acceptable range, a reasonable number of iterations and detection data are adopted in numerical experiments.

**Figure 3:**
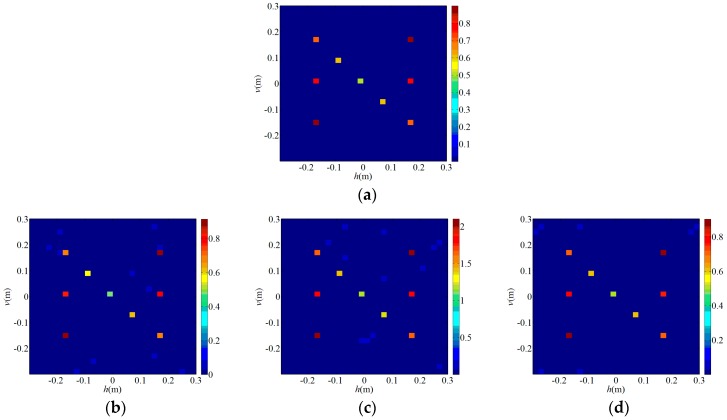
Reconstruction results for sparse target: (**a**) the original sparse target; (**b**) orthogonal matching pursuit (OMP) algorithm for TCAI; (**c**) truncated Wirtinger flow (TWF) algorithm for PTCAI; (**d**) Wirtinger flow with optimal stepsize (WFOS) algorithm for PTCAI.

**Figure 4:**
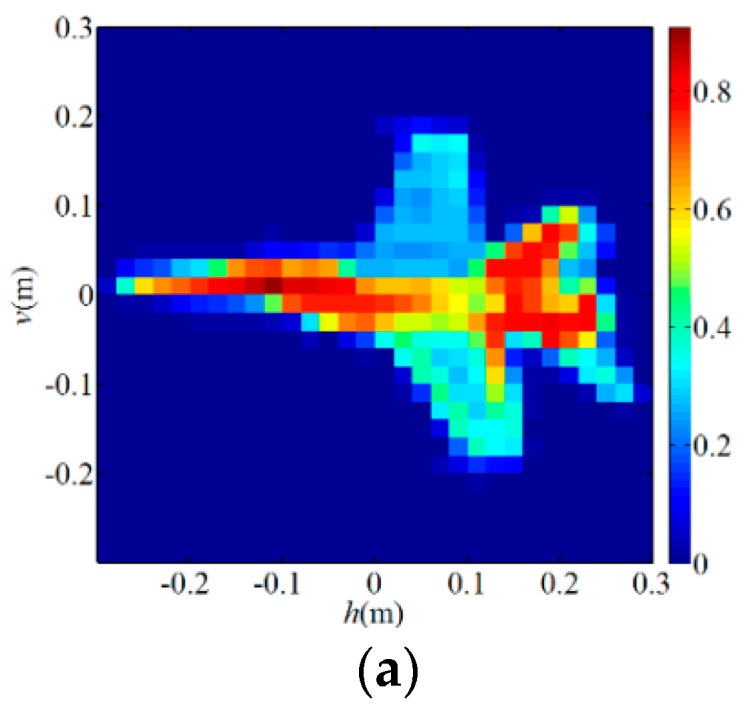

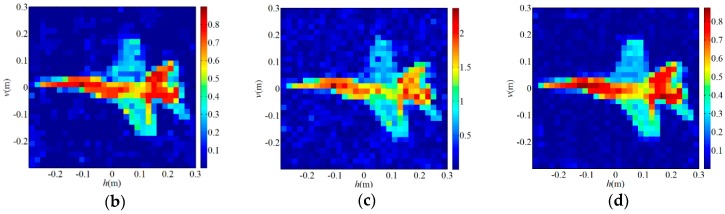
Reconstruction results for extended target: (**a**) the original extended target; (**b**) TVAL3 algorithm for TCAI; (**c**) TWF algorithm for PTCAI; (**d**) WFOS algorithm for PTCAI.

Figure 3a (*T/W* = 1/100) and Figure 4a (*T/W* = 4/9) are original images of the sparse target and the extended target, respectively, and the rest are reconstruction results of each algorithms. Figure 3b is a TCAI result of the OMP algorithm for the sparse target while Figure 4b is a TCAI result of the TVAL3 algorithm for the extended target, where the targets are both reconstructed successfully. Although Figure 3b and Figure 4b have a few spurious weak scatterers due to the influence of noise, strong scatterers are obviously recovered in both of them and the edges of targets look clear. Figure 3c and Figure 4c represent the reconstruction results of the TWF algorithm for the PTCAI system, both with some spurious scatterers and blurred target edges. This is mainly because the number of detection data *L* is not sufficient for the TWF algorithm and the noise also affects the imaging quality. In fact, as *L* increases, the reconstruction accuracy of the TWF algorithm for the target can be improved significantly. It should be noted that in order to obtain better performance, the TWF algorithm should change its step size α due to various experimental conditions and the step size α is an empirical value. Thus, the TWF algorithm may not be suitable for the actual PTCAI system and variable experimental conditions.

As can be seen from Figure 3d and Figure 4d that the PTCAI system with the WFOS algorithm can successfully reconstruct the targets when only detecting the intensity information of the echo signal, whether it is sparse or extended. Although imaging quality is affected by noise, all strong scatterers are recovered and the target edges are clear. In addition, since the WFOS algorithm uses the optimal step size in each iteration, it does not need to set empirical values and is suitable for the actual PTCAI system and variable experimental conditions (only $\alpha_{y}=3$, which is the parameter needed for truncated initialization and does not affect the iteration result). To further verify the conclusions, the runtime of algorithm, RIE, and PSI are recorded when TCAI and PTCAI reconstruct the extended target, and the results are shown in Table 2. As for extended targets, the PTCAI system based on the WFOS algorithm has almost the same PSI and RIE compared with the TCAI system based on the TVAL3 algorithm, which can accurately reconstruct the target. However, the shortcoming of PTCAI is that the WFOS algorithm requires an excessive amount of detection data and a long runtime. But this cannot hinder the development of PTCAI. Using an incoherent detector array instead of a single incoherent detector will be able to reduce the encoding and sampling times while the development of phase retrieval algorithms will decrease the demand for the number of detection data, which can effectively reduce imaging time.

### 4.2. Analyses of the Number of Detection Data for PTCAI with Single Incoherent Detector

In the previous section, performance of the PTCAI system with the WFOS algorithm is demonstrated to be as good as the TCAI system with same simulation settings, but since the WFOS algorithm uses spectral methods to solve non-convex problems iteratively, it requires excessive detection data. In order to study the influence of the number of detection data for PTCAI, we perform a numerical experiment for an extended target only by changing the number of detection data while adopting the same parameters and system conditions for the WFOS algorithm mentioned above. The simulation setting is SNR = 20 dB.

**Figure 5:**
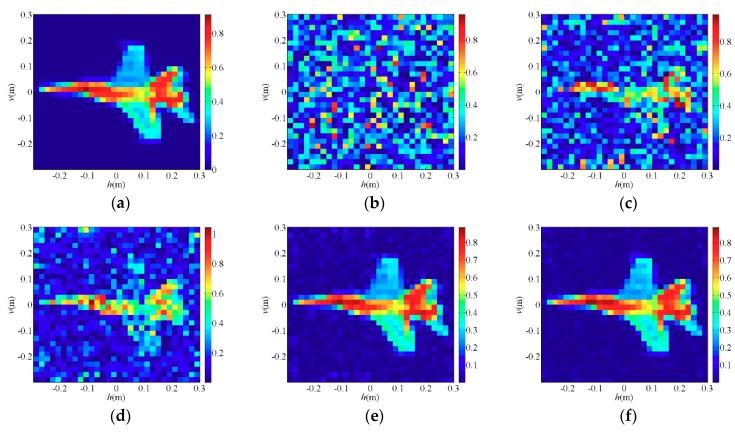
Reconstruction results for different number of detection data in the PTCAI system based on WFOS algorithm: (**a**) the original extended target; (**b**) *L/W* = 1; (**c**) *L/W* = 3; (**d**) *L/W* = 3.5; (**e**) *L/W* = 5; (**f**) *L/W* = 10.

Figure 5a is the original extended target, and the ratio between the target size (*T*) and the number of grid cells (*W*) is *T/W* = 4/9. Figure 5b–f show reconstructed results of PTCAI with the WFOS algorithm, where the ratio between the number of detection data (*L*) and the number of grid cells (*W*) is *L/W* = 1, *L/W* = 3, *L/W* = 3.5, *L/W* = 5, *L/W* = 10, respectively. Figure 5b–d show the failure to reconstruct the target, and all of them have many spurious scatterers that make it impossible to distinguish the shape of the target clearly. Figure 5e,f are high quality imaging results with a few spurious scatterers that caused by noise. It can be seen from Figure 5 that as the ratio of *L/W* increases, the imaging quality of PTCAI is gradually enhanced, and the reconstructed target can be successfully obtained when the ratio of *L/W* reaches about 5.

**Figure 6:**
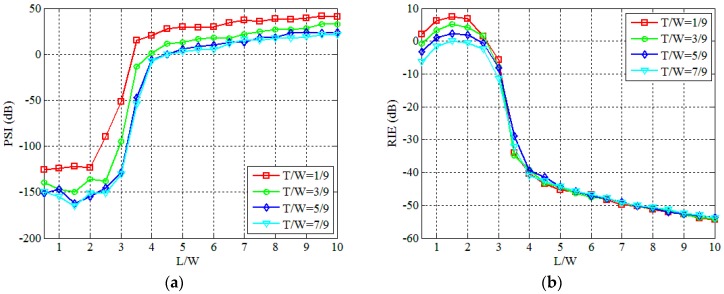
Influence of *L* in the PTCAI system based on WFOS algorithm: (**a**) PSI; (**b**) RIE.

Although the quality of imaging can be better and more stable with more detection data, excessive detection data inevitably increase the computation complexity and imaging time, and thus decrease the frame rate. Figure 6a,b show the changing trend of PSI and RIE for different sizes and shapes of targets, respectively. In Figure 6, each curve represents the average results over 50 Monte Carlo trials and shape of the target changes randomly in each trial. As can be seen from Figure 6, for different sizes and shapes of targets, the PSI and RIE have significant changes from *L/W* = 2 to *L/W* = 4. When PSI ≥ 0, it can been found that *L/W* = 3.5 for the target size *T/W* = 1/9 and *L/W* = 5 for the target size *T/W* = 7/9, respectively. Therefore, the larger the target size, the more detection data is required for successful imaging. As the ratio of *L/W* increases, RIE gets smaller and smaller while imaging results can become better and better. Thus, it is possible to find the balance between computation complexity and imaging quality for the PTCAI system with an optimal ratio of *L/W* from Figure 6.

**Figure 7:**
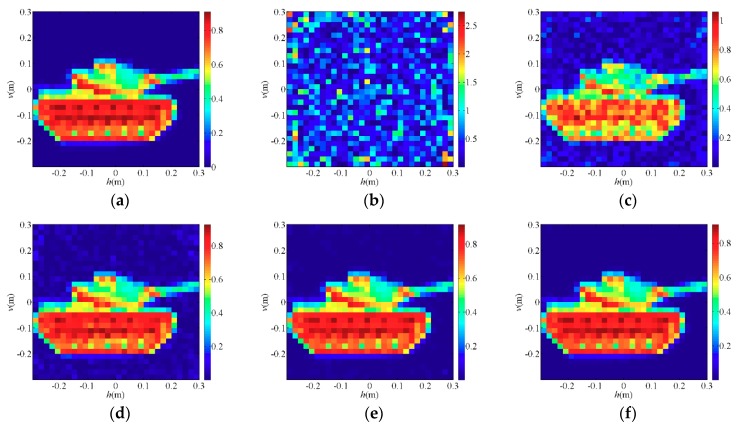
Imaging results for different SNRs in the PTCAI system based on the WFOS algorithm: (**a**) the original extended target; (**b**) SNR = 0 dB; (**c**) SNR = 10 dB; (**d**) SNR = 20 dB; (**e**) SNR = 30 dB; (**f**) SNR = 40 dB.

**Figure 8:**
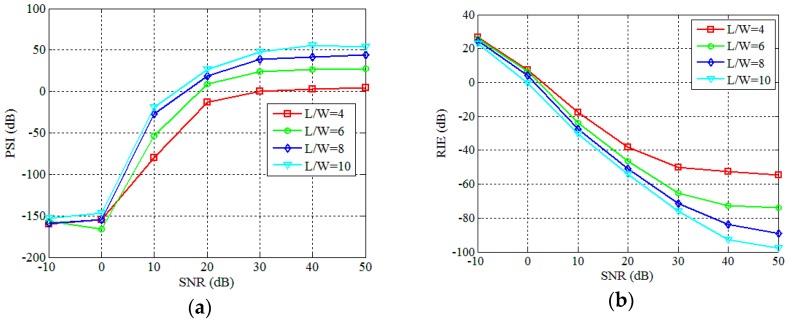
Influence of different SNRs in the PTCAI system based on the WFOS algorithm: (**a**) PSI; (**b**) RIE.

Next, we simulate the reconstruction performance of the PTCAI system based on the WFOS algorithm at different SNRs. Figure 7a is the original extended target that the ratio of *T/W* is 5/9. When *L/W* = 10, Figure 7b–f are results of reconstructing the target in SNR = 0 dB, SNR = 10 dB, SNR = 20 dB, SNR = 30 dB and SNR = 40 dB, respectively. Due to the low SNR, the target reconstruction in Figure 7b fails and most of the strong scatterers are not accurately recovered in Figure 7c. Figure 7d,e show that all of reconstructions are successful and the image quality is improving with the increase of SNR. In Figure 8, we evaluate the performance of the PTCAI system based on the WFOS algorithm in different SNRs conditions with the setting *T/W* = 5/9 and each curve represents the average results over 50 Monte Carlo trials. The SNR varies from −10 dB to 50 dB while the *L/W* ratios that can guarantee successful imaging in contrast experiments are chosen to be 4, 6, 8 and 10, respectively. From Figure 8, it can be seen that the PTCAI system with the WFOS algorithm has inferior performance in low SNR conditions. But, with the SNR increasing, the PSI gradually increases and the reconstruction of the target can be finally successful when PSI ≥ 0. Besides, as the SNR increases, the RIE decreases rapidly and the imaging quality increases rapidly. The results in Figure 8 also demonstrate that imaging quality can be improved by increasing the ratio of *L/W* under the condition that the SNR is constant, which is consistent with our previous conclusions.

### 4.3. Parameter Analyses of the Incoherent Detector Array

As presented in the end of Section 2.2 and Section 4.1, the use of the incoherent detector array can significantly reduce the encoding and sampling times without degrading the imaging quality. In this section, we replace the single incoherent detector with an incoherent detector array and focus on analyzing different element spacing of the incoherent detector array. The incoherent detector array contains M=e×e array elements and the spacing between two adjacent elements is *Da*. The simulation setting is SNR = 20 dB and *T*/*W* = 5/9, and each curve of the results represents the average results over 50 Monte Carlo trials. Other parameters and system conditions are the same with Table 1.

**Figure 9:**
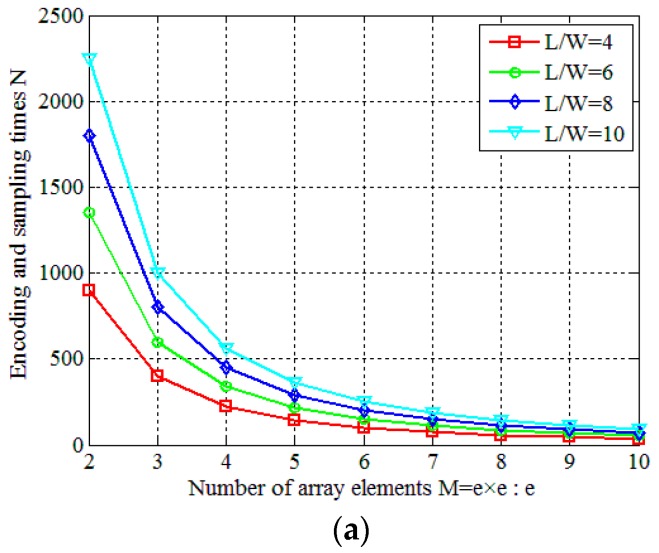

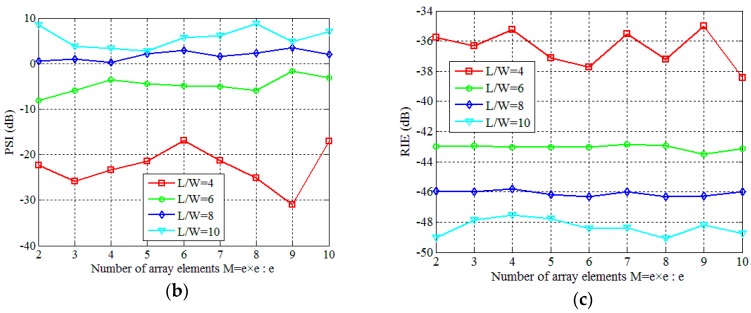
Influence of the number of array elements M=m×m in the PTCAI system based on the WFOS algorithm: (**a**) encoding and sampling times *N*; (**b**) PSI; (**c**) RIE.

**Figure 10:**
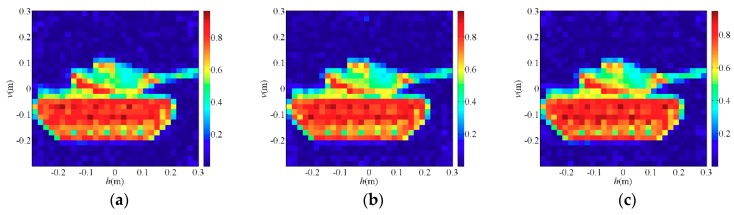
Reconstruction results for different M=e×e in the PTCAI system based on the WFOS algorithm: (**a**) M=2×2; (**b**) M=4×4; (**c**) M=7×7.

Firstly, the element spacing is set to *Da* = 0.002 m, and the number of array elements increases from M=1×1 to M=10×10. The final results obtained by the WFOS algorithm are shown in Figure 9. Obviously, as shown in Figure 9a, at different ratios of *L*/*W*, the encoding and sampling times *N* sharply decrease as *M* increases. As shown in Figure 9b,c, when the ratios of *L*/*W* are 8 and 10, both of them satisfy the conditions for successful reconstruction. When we use the incoherent detector array which contains *M* array elements, the PSI and RIE change slightly while the imaging quality does not change significantly. In Figure 10a−c, all ratios of *L*/*W* are 8 and RIEs are −46 dB, −45 dB and −46 dB, respectively. Although PSI and RIE fluctuate with *M*, they are all within reasonable limits where the PTCAI system can successfully reconstruct the target, as shown in Figure 10.

**Figure 11:**
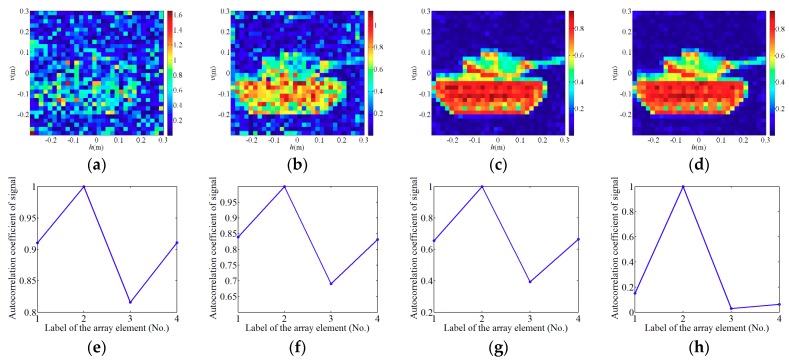
(**a**–**d**) Reconstruction results of the PTCAI system with *Da* = 0.0001 m, *Da* = 0.00014 m, *Da* = 0.0002 m and *Da* = 0.001 m, respectively; (**e**–**h**) correlation of signals received by different array elements when *Da* = 0.0001 m, *Da* = 0.00014 m, *Da* = 0.0002 m and *Da* = 0.001 m, respectively.

**Figure 12:**
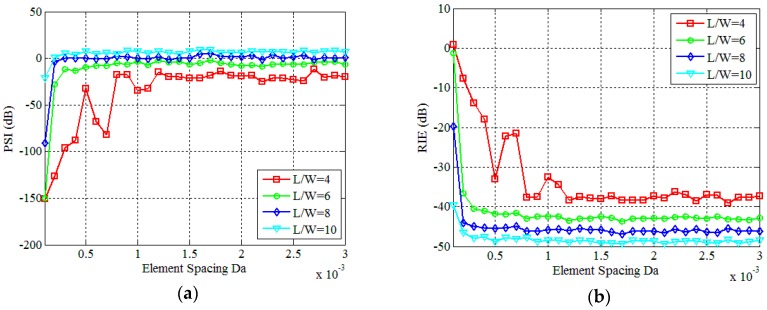
Influence of different *Da*: (**a**) PSI; (**b**) RIE.

Then, we fix the number of array elements to M=2×2 and gradually increase the element spacing from 0.0001 m to 0.003 m according to the interval of 0.0001 m. The final results obtained by the WFOS algorithm are shown in Figure 11 and Figure 12. Among them, at the ratio of *L*/*W* is 8, Figure 11a–d are reconstruction results of different element spacing *Da*, and Figure 11e–h are results of correlation processing between the signals received by the array elements No.1, No.3, and No.4 and the signals received by the array elements No.2, respectively. Obviously, Figure 11 shows that as *Da* gradually increases, the non-correlation of the signals between the various array elements becomes ever stronger, and the imaging quality of PTCAI becomes ever higher. This proves that the more uncorrelated signals detected by each array element, the less correlated signals finally obtained by the array, which facilitates the accurate solution of the imaging equation and improves the reconstructing accuracy. It can be seen from Figure 12, PSI and RIE are finally stabilized as the distance between the array elements increases. In particular, it can be concluded that there is a minimum distance between each array element for the proposed PTCAI system. If the distance is smaller than this minimum, the intensity information detected by different array elements will be quite similar to each other, just like that detected by a single detector, so that the encoding and sampling times *N* cannot be reduced.

## 5. Conclusions

In this paper, we propose a PTCAI method that can achieve high resolution, high frame rate and is forward looking based on detecting the intensity information incoherently. Firstly, mathematical imaging models of the PTCAI system with the single incoherent detector and the incoherent detector array are built in detail, and the target reconstruction principle and algorithms are introduced to reconstruct the original target with high resolution. Secondly, according to the results of a numerical experiment, we have demonstrated that the proposed PTCAI system can reconstruct sparse and extended targets as accurately as the conventional TCAI system. Moreover, the number of detection data plays a crucial role on the reconstruction accuracy and imaging time of the PTCAI system. Finally, using an incoherent detector array instead of a single incoherent detector can effectively reduce the encoding and sampling times *N* and shorten imaging time without sacrificing imaging quality. In conclusion, this paper proves the feasibility of the PTCAI system and provides positive guidance for studying and designing this novel method, and the proposed PTCAI method has many potential applications such as security checks, terminal guidance, and battlefield reconnaissance etc.

## Figures and Tables

**Table 1:** Basic parameter settings used in the simulations.

Parameter	Value
Center frequency	340 GHz
Pulse width	100 μs
Bandwidth	12 GHz
Imaging distance	10 m
The size of the coded aperture	0.5 m×0.5 m
The number of elements in the coded aperture	30×30
The size of the grid cells in the imaging plane	0.02 m×0.02 m
The number of grid cells in the imaging plane	30×30
The distance between the coded aperture and the receiver	0.1 m

**Table 2:** Comparison of receiving methods and algorithms.

Detecting Mode	Algorithms	Probability of Successful Imaging (PSI) (dB)	Relative Imaging Error (RIE) (dB)	Number of Detection Data	Runtime (s)
Coherent detection (TCAI)	TVAL3	9.825	−35.931	450	0.279
Incoherent detection (PTCAI)	WFOS	10.691	−44.131	4500	1.463
Incoherent detection (PTCAI)	TWF	0.134	−12.653	27,000	40.466
